# Degree of conversion and depth of cure of Ivocerin containing photo-polymerized resin luting cement in comparison to conventional luting agents

**DOI:** 10.12669/pjms.342.14491

**Published:** 2018

**Authors:** Fahad Alkhudhairy, Abdulaziz AlKheraif, Mustafa Naseem, Rawaiz Khan, Fahim Vohra

**Affiliations:** 1 Fahad Alkhudhairy, Department of Restorative Dental Sciences, College of Dentistry, King Saud University, Riyadh, Saudi Arabia; 2 Abdulaziz AlKheraif, Dental Health Department, College of Applied Medical Sciences, King Saud University, Riyadh, Saudi Arabia; 3 Mustafa Naseem, Department of Preventive Dental Sciences, Dar Al Uloom University Al Mizan St, Al-Falah, Riyadh, Saudia Arabia; 4 Rawaiz Khan, College of Dentistry Research Center (CDRC), College of Dentistry, King Saud University, Riyadh, Saudi Arabia; 5 FahimVohra, Department of Prosthetic Dental Science, College of Dentistry, King Saud University, Riyadh, Saudi Arabia

**Keywords:** Degree of Conversion, Ivocerin, Luting cements, Micro hardness

## Abstract

**Objective:**

To evaluate the degree of conversion (DC) and depth (extent) of cure of four resin cements (Variolink E, Calibra, NX3 and Variolink N) using Fourier transform infrared (FTIR) and Vickers Micro hardness (MH).

**Methods:**

Ten disks (1mmx2mm) of each resin cement were light cured through a ceramic disk for 40 seconds prior to assessment. The ATR spectra of the uncured resin were collected in absorbance mode from 16 scans at 4 wave number resolutions. Degree of conversion was calculated by estimating the changes in peak height ratio of the absorbance intensities of aliphatic C=C peak at 1638 cm^-1^ and that of an internal standard peak of aromatic C=C at 1608 cm^-1^ during polymerization. For Vickers microhardness testing 10 disks of each cement specimen was exposed to 100 grams of load for 15 seconds. Three indentations were made 0.5mm apart and an average Vickers micro-hardness (MH) for each specimen. Two way ANOVA and multiple comparison tests were performed to assess data.

**Results:**

The highest degree of conversion by peak area was shown by Variolink-Esthetic [light-cure (87.18±2.90%)]; however the lowest was observed in samples of Variolink-N [Dual cure (44.55±4.33%)]. Similarly, Variolink-Esthetic and NX3 cement showed significantly higher MH as compared to other groups.

**Conclusion:**

Ivocerin containing Variolink–E cement showed high degree of conversion and extent of polymerization when compared to conventional light and dual cure luting cements.

## INTRODUCTION

Advancement in resin based dental materials including resin luting cements is at the center of developing predictable adhesive dentistry. Properties of resin based cements critical to the success of adhesive restorations include adequate bond strength, low solubility, biocompatibility, color stability, low film thickness and polymerization shrinkage.^1^ Resin cements are classified according to the filler type (micro, micro, nano), mode of activation (photo, chemical and dual cure) and bonding mechanism (self-etch and total etch).^2^ Resin cements contain different monomers linked together during polymerization reaction. The characteristics and properties of resin based cements is dependent on the degree of conversion of monomers to polymers.^3^ Inadequate degree of conversion compromises the mechanical properties of the cement effecting bond stability and strength and therefore clinical longevity.^4^ Furthermore, it increases the risk of water-sorption by the material leached or unreacted monomer which may cause irreversible pulpal damage and allergic reaction.^5^ Alternatively, if the conversion of monomer to polymer is maximized the possibility of polymerization shrinkage of the filling material is increased.^6^

Dual cure cements, utilize both light and self-curing chemical reaction to produce high number of radicals and achieve high degree of conversion from monomer to polymer. However, dual cure cements contain tertiary amines for initiation of polymerization which are implicated in compromising their color stability.^7^ By contrast, photo-polymerized resin cements use Norrish Type-II photo initiator (camphorquinone) to induce free radical formation resulting in polymerization reaction.^6^ In addition, photo-polymerized cements show better color stability (as no tertiary amines are used as chemical activator) and have ambient working time.^8^ However degree of polymerization for photopolymerized cements depends on light penetration and exposure, which is influenced by the thickness and translucency of adhesively bonded restorations.^9^ Many attempts have been made to improve the degree of conversion and depth of cure for photopolymerized cements by improvements in size of the filler particle, color, incremental technique, saturation, shades, photo initiator, number of firing cycles and distance of light curing tips.^10^

One such example is the incorporation of Ivocerin and thiocarbamide in photopolymerized resin cements as initiators.^11^ It is claimed, that Ivocerin(Variolink Esthetic, Ivocalr Vivadent) when exposed to light results in a cleavage of chemical bond within the photo initiator itself, which reacts with monomer to form polymerization network.^11^ Work documented by Jerri et al.,^12^ and Ilie^13^ Moszner et al.^14^, claims Ivocerin as a photo initiator have faster, greater polymerization at depth, superior reactivity to curing light having a broad wave length range of 370nm to 460nm compared to camphorquinone).

The success of resin cement is reliant on the optimal cure of the ceramic restoration. To our knowledge from indexed literature, there is no evidence on degree of Conversion and extent of polymerization of Variolink E (having Ivocerin as initiator) with other Dual cure cements (Variolink N, Calibra and NX3). It is hypothesized that Variolink E having Ivocerin as photo initiator will have better degree of conversion and depth of cure compared to other luting cements. Therefore, the objective of the study was to evaluate the degree of conversion of all four resin cements using Fourier transform infrared (FTIR) and the depth of cure or extent of polymerization using Vickers Microhardness.

## METHODS

The degree of conversion and depth of cure of four different resin based cements were assessed using Fourier transform Infrared (FTIR) spectroscopy and Vickers micro-hardness methods. The composition of the resin cements included in the study is presented in Table-I.

**Table-I:** Materials used in the study.

Product	Polymerization	Initiator	Content	Manufacturer
Variolink- Esthetics (LC)	Light cure	Ivocerin and thiocarbamide hydroperoxide self-curing initiator	UDMA Urethane dimethacrylate monomers, filler, initiator and stabilizers, pigments	Ivoclar-Vivadent
Calibra	Dual Cure	Benzoyl peroxide Camphorquinone	BISGMA resins monomer, glass fillers, coupling agent, peroxide	Dentsply Caulk
NX3 (LC)	Light cure	Proprietory redox initiator system	HEMA, uncured methacralayte, titanium dioxide pigments	Kerr, Canada
Variolink –N (high viscosity)	Dual Cure	Tertiary amine camphorquinone	BISGMA Barium glass filler, Di-methacrylates, silica, initiators, stabilers, pigments	Ivoclar-Vivadent

### Degree of Conversion

The degree of conversion among four cements (Calibra, NX3, Variolink-N and Variolink-E) (n=10) were calculated using Fourier transform Infrared (FTIR) spectroscope (Model: 4100 Jasco Corporation Tokyo Japan) along with Attenuated total reflectance unit (Pike miracle ATR, Diamond ZnSeW technology). The ATR spectra of the uncured resin were collected in absorbance mode from 16 scans at 4 wave number resolutions. Background spectra were collected through an empty mold with one glass slide to avoid internal reflectance patterns prior to running the samples. Vinyl molds with (1mm thickness x 2mm inner diameter) were used to place resin cements. These vinyl molds were surrounded by Teflon molds. To guarantee evenness of the specimens, inhibition of polymerization by oxygen and easy placement of ceramic disc (Lithium Disilicate, IPS Emax, Ivoclar Vivadent- 1mm) on resin cement Mylar strips (DuPont Mylar 0.002 gauge/60mm thick) were used. With the help of a Teflon ring, the tip of light curing unit (Woodpecker I-LED dental wireless LED curing light Curing 2300 mw/CM2) and ceramic disc (Lithium Disilicate, IPS Emax, Ivoclar Vivadent- 1mm) were positioned. The light cure resin cements (Variolink-E and NX3) were then exposed for 40 seconds by keeping the LED tip at exactly on the same diameter as that of disc sample. The experimental study was in in accordance with the CRIS (Checklist for Reporting In-vitro Studies) guidelines.

Similarly, for dual cured cements (Calibra and Variolink-N) equal amount of base and catalyst pastes were mixed in accordance with the manufacturer instructions. After mixing the base and catalyst, the cement was allowed to auto-polymerize (self-cure) for two minutes. All the dual cured cements were then light cured through the ceramic disc (Lithium Disilicate, IPS Emax, Ivoclar Vivadent- 1mm) using a quartz tungsten halogen (Woodpecker I-LED dental wireless LED curing light Curing 2300 mw/CM2) for forty seconds. The specimens were placed inside the FTIR chamber and cured spectra were collected.

Degree of conversion was calculated by estimating the changes in peak height ratio of the absorbance intensities of aliphatic C=C peak at 1638 cm^-1^ and that of an internal standard peak of aromatic C=C at 1608 cm^-1^ during polymerization, in relation to the uncured material. DC % for each specimen was calculated using the following equation:


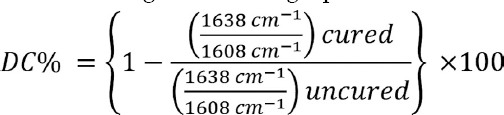


### Micro hardness testing

For microhardness test, 10 specimens in each cement group (Calibra, NX3, Variolink-N and Variolink-E) were fabricated. Specimens were prepared using a Teflon mold (2.0mm in thickness and 5mm in diameter), which was covered by a polyester strip. Light cure resin cements (Variolink-E and NX3) were dispensed in the mold and covered with a mylar strip and 1mm ceramic disk (Lithium Disilicate, IPS Emax, Ivoclar Vivadent- 1mm) prior to photo-polymerization. Similarly, for dual cured cements (Calibra and Variolink-N) equal amount of base and catalyst pastes were mixed in accordance with the manufacturer instructions. After mixing the base and catalyst, the cement was placed into the mold and allowed to auto-polymerize (self-cure) for two minutes. Dual cured cements were then light cured through the ceramic disc (Lithium Disilicate, IPS Emax, Ivoclar Vivadent- 1mm) using a quartz tungsten halogen (Woodpecker I-LED dental wireless LED curing light Curing 2300 mw/CM2) for forty seconds. For Vickers microhardness testing 100 grams of load was applied for 15 seconds. Three indentations were made on the surface of each specimen randomly using Vickers Hardness tester (HMV-2 Shimadzu Corp). Each indentation was separated by 0.5mm. The three values were averaged to give single Vickers hardness (VH) for each specimen.

### Statistical Analysis

A two way ANOVA was used to analyze data of Vickers micro hardness testing for the depth of cure and Degree of Conversion DC. All post hoc multiple comparison tests were performed using Tukey test. Statistical significance was set as 0.05.

## RESULTS

### Degree of Conversion (DC)

**Fig.1:**
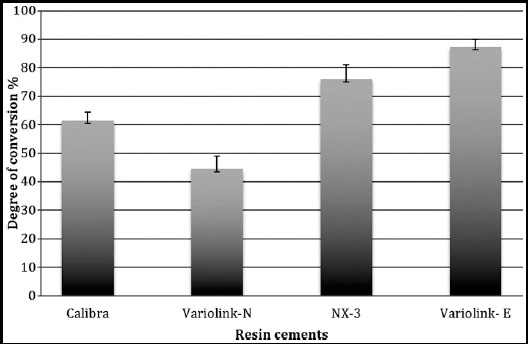
Degree of conversion (%) among different study groups.

The highest degree of conversion by peak area was shown by photopolymerized samples of Variolink-Esthetic [light-cure (87.18±2.90%)], however the lowest was observed in samples of Variolink-N [Dual cure (44.55±4.33%)] (Table-II and Fig. 1, 2 & 3). The assessed degree of conversion for Calibra and NX3 cements was 61.52±3.87% and 75.91±5.11% respectively.

**Table-II:** Means (SD) for degree of conversion (%) values among study groups using ANOVA and Tukey multiple comparisons test.

Cement Type	Mean*	SD	SE	Variance	P-value^!^
Calibra	61.525^A^	3.876	2.215	10.851	<0.001
Variolink-N	44.556^B^	4.336	2.255	9.882	
NX-3	75.919^C^	5.113	2.416	10.333	
Variolink- E	87.185^D^	2.906	1.678	8.448	

* Dissimilar alphabets in the same column means statistical significant difference (Tukey multiple comparison test)

! Showing significant difference among study groups (ANOVA).

**Fig.2:**
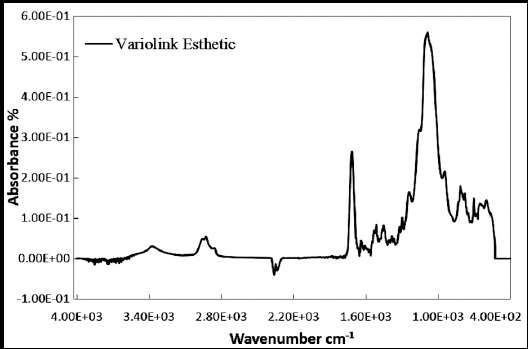
FTIR graph showing heights of aliphatic C=C peaks for Variolink Esthetic cement.

**Fig.3:**
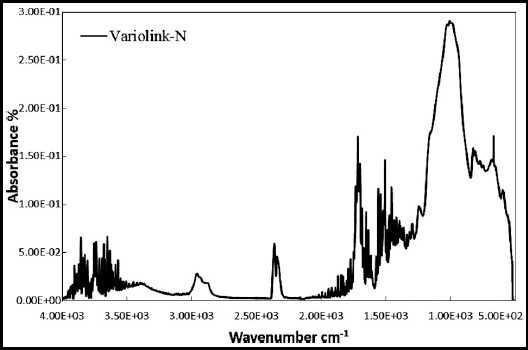
FTIR graph showing heights of aliphatic C=C peaks for Variolink-N cement

The degree of conversion among the cements compared in the study was significantly different (ANOVA, P<0.01) (Table-II). Degree of conversion for samples for Variolink-Esthetic was significantly higher than all other cements (Tukey multiple comparisons test- Table-II). NX3 samples showed degree of conversion greater than Variolink-N and Calibra cements (p<0.01). Calibra samples showed significantly lower degree of conversion compared to all other cement groups (Table-II).

### Vickers Micro-hardness (MH)

The means and standard deviations for MH are presented in Table-III. The highest and lowest MH value among resin cements polymerized through ceramic disk was shown by Variolink-Esthetic (47.71±1.01 VHN) and Variolink-N (33.70±0.78 VHN) respectively (non-thermocycled). Luting cements, namely Calibra and NX3 showed MH values of 37.36±1.2 VHN and 44.75±0.83 VHN respectively (non-thermocycled). MH among thermocycled samples also showed similar pattern, with Variolink–Esthetic having highest and Variolink-N showing the lowest values.

**Table-III:** Means (SD) for Vickers micro-hardness (VHN) values among study groups before and after ageing using ANOVA and Tukey multiple comparisons test.

Cement Type	Control		Thermocycled		P value^!^

	Mean *	SD	Mean	SD	
Calibra	37.36^Aa^	1.29	32.75^Ab^	0.34	<0.001
Variolink-N	34.70^Ba^	0.78	30.95^Ab^	0.61	
NX-3	44.74^Ca^	0.83	44.05^Ba^	0.28	
Variolink- E	47.71^Da^	1.01	46.69^Ba^	0.35	

Dissimilar capital alphabets in the same column are significantly different.

Dissimilar small alphabets in the same row are significantly different.

**Fig.4:**
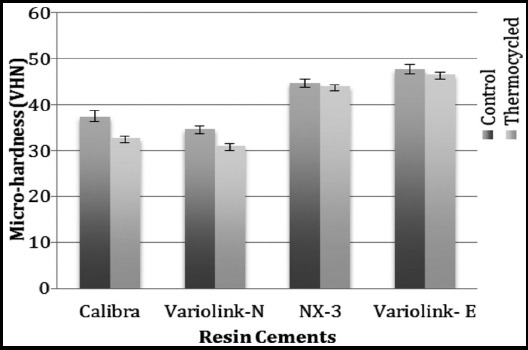
Micro-Hardness values (VHN) among different study groups before and after thermocycling.

MH values among non-thermocycled samples of different resin cements were significantly different (ANOVA, p<0.01, Fig.4). Ageing with the help of thermocycling significantly reduced MH for Calibra and Variolink-N respectively. However samples of NX3 and Variolink –Esthetic cements, showed comparable outcomes of MH, with and without thermocycling (Table-III & Fig.2). Following thermocycling, samples in Variolink-Esthetic and NX3 cement showed significantly higher MH as compared to other groups. Although MH values after thermocycling in Calibra and Variolink-N samples were comparable, MH for these groups was statistically lower than NX3 and Variolink –Esthetic study groups respectively.

## DISCUSSION

The present study was based on the hypothesis that Variolink E having Ivocerin as photo initiator will have better degree of conversion and extent of polymerization to other luting cements(Variolink N, Calibra and NX3). Intrestingly, this hypothesis was accepted as Variolink E had better degree of conversion and depth of cure.

Variolink E having patent Ivocerin as a photoinitiator was compared with (NX3) light cure and two dual cure cements (Variolink N and Calibra). The degree of conversion was found to be comparable among both light cure cements Variolink E (87.18±2.90) and NX3 (75.91±5.11). Further, the highest degree of conversion was found to be of Variolink E (87.18±2.90). Degree of conversion is important as it governs the physical and mechanical properties of luting cements such as compressive strength, tensile strength, hardness, toughness and biocompatibility; and is directlty related to the monomer conversion during polymerization.^4^ Inadequate curing with reduced degree of conversion alters bond strength and dimensional stability; and may result in possible allergic reactions compromising clinical performance of the luting cement.^15^ However, there are various methods to assess extent of polymerzation of luting cement i.e. physical determination of surface hardness, Diffrential scanning calorimetry (DSC) and mid-infra red spectroscopy (mid-IR). In the present study we used microhardness test to investigate extent of polymerization as it is considered more sensitive in assessment of post-mix polymeriztion.^16^ Secondly, degree of conversion was tested using Fourier transform Infrared (FTIR) spectroscopy due to its ability of rapid scanning, high wavelength, better resolution, stability and accuracy.^17^ To mimic the clinical situation, a ceramic disc (1 mm) was placed over the luting cements, the thickness and translucency of which are critical to the degree of conversion of cements, particularly photo-polymerized cements.

Ivocerin is a Norish Type-II photo initiator, which when exposed to light cleavages a chemical bond within itself and reacts with monomer to form polymerization network. Ivocerin along with camphorquinonein (Tetric Evoceram) dual cure bulk fill composites has shown significant improvement in degree of conversion. Studies by Al-Mansour et al.^18^, Yap et al.^19^, Rueggeberg et al.^11^ have shown Ivocerin acts as a polymerization booster in bulk fill composites, showing high reactivity to curing light, and allowing efficient polymerization at the depth of 4mm. The findings of these studies by Yap et al.^20^, Almansour et al.^18^, Rueggeberg et al.^11^ are in agreement with the present study showing degree of conversion of samples for Variolink-Esthetic significantly higher than other cements. Similarly, outcomes for NX3 samples showed degree of conversion greater than Variolink-N and Calibra cements (p<0.01). A plausible reason for better result of NX3 is the redox initiator system which is free from tertiary amine and benzoyl peroxide.^21^ This redox initiator system resolves incompatibility issues with acidic adhesives, making NX3 compatible with total and self-etch adhesives.^22^ Furthermore, Calibra and Variolink-N samples showed significantly lower degree of conversion compared to NX3 and Variolink-E. A conceivable clarification for this outcome may be related to the instability of Benzoyl peroxide (BPO) and tertiary amine as initiator in these dual cure cements.^23^

Micro Hardness (MH) among (non-thermocycled) samples showed similar pattern, with Variolink–Esthetic having highest and Variolink-N showing the lowest values. Following thermocycling, samples in Variolink-Esthetic and NX3 cement showed significantly higher MH as compared to other groups. Multiple factors influence MH of resin materials including, monomer conversion, filler content, thickness of ceramic, type of luting materials and time of assessment.^20^ Available evidence advocates that the value of MH can be independent from degree of conversion if there is a difference in the monomer content of cement.^8^ This could be a probable reason for low MH scores in Claibra and Variolink- N, as both these luting cement contain similar monomer [bisphenol A-glycidyl methacrylate (BISGMA)]. Whereas, Variolink-N and NX3 are comprised of urethane-dimethacrylate monomer (UDMA) and Hydroxyethyl methacrylate (HEMA) respectively. MH is considered to be more sensitive, reliable way of assessing conversion of monomer to polymer after twenty-four hours of polymerization initiation.^24^ However in the present study, assessment of MH was performed after fifteen minutes of polymerization initiation; therefore the outcomes may not be a true reflection of the MH scores of different luting cements. Similarly, for light cure cements stiffening might take place when exposed to curing light.^25^ Although the material seems hard but it should be noted that polymerization reaction continues for twenty-four hours. Therefore, clinically it is recommended to delay finishing and polishing for at-least twenty-four hours for better desirable outcomes.^20^ Although Variolink-E (Ivocerin) showed high DC and MH values in the present study, its clinical effectiveness over other cements cannot be based on these outcomes only. Other factors such as color stability, bond strength, wear resistance; handling properties contribute immensely in the clinical success of resin cements. Therefore, further studies comparing the color stability and durability of cements containing Ivocerin are recommended.

In the present study MH, scores were measured after fifteen minutes. Evidence suggests that since monomer to polymer is a continues reaction and if MH scores are calculated early there will be lack of detectable polymers.^25^ Therefore, it will be interesting to evaluate the MH and DC scores of Variolink Esthetics having Ivocerin as photoinitiator over a period. In addition, previous studies have shown a correlation between degree of conversion and polymerization shrinkage among resin cements.^6^ Hence, additional studies are suggested to assess and correlate the degree of conversion of Variolink Esthetics to polymerization contraction in clinically relevant settings.

## CONCLUSION

Within the limitations of the study, Ivocerin containing Variolink–E cement showed high degree of conversion and extent of polymerization when compared to conventional light and dual cure luting cement

### Authors’ Contribution

**FV:** Data collection, study design, manuscript writing, final manuscript approval.

**MN:** Data collection, study design, manuscript drafting, data analysis, manuscript approval.

**FA:** Data collection, manuscript approval and data interpretation.

**AA:** Data collection, writing, revise, editing and final manuscript approval.
